# Psychopathy in adult male offenders from Latin America: an item response theory analysis of the self-report psychopathy-short form (SRP-SF)

**DOI:** 10.3389/fpsyg.2025.1684621

**Published:** 2026-01-06

**Authors:** Nicolás Trajtenberg, Olga Sánchez de Ribera, Elizabeth León-Mayer, Pablo Menese, Craig S. Neumann

**Affiliations:** 1 Department of Criminology, School of Social Science, The University of Manchester, Manchester, United Kingdom; 2 The University of Manchester School of Social Sciences, Manchester, United Kingdom; 3 Department of Psychiatry, School of Medicine, National University of La Plata, La Plata, Buenos Aires, Argentina; 4 Universidad de la Republica Uruguay- Centro Universitario de Tacuarembo, Montevideo, Uruguay; 5 Department of Psychology, Texas State University, San Marcos, TX, United States

**Keywords:** psychopathy, item response theory, SRP-SF, offenders, Latin America

## Abstract

**Introduction:**

Research on the item properties of the Psychopathy Checklist–Revised (PCL-R) is extensive, and the Self-Report Psychopathy Scale-Short Form (SRP-SF) is a derivative of the PCL-R. To date, studies on the SRP-SF items have primarily relied on classical test theory with Western populations, although research using latent variable models across different cultures is emerging.

**Methods:**

This study applied an Item Response Theory approach using the Graded Response Model to examine SRPSF item properties in two incarcerated male samples from Chile (N = 208) and Uruguay (N = 331). It also examined the item functioning differences between these two samples.

**Results:**

Findings indicated that Facet 1 (Interpersonal) was the most informative for measuring the latent psychopathy trait. However, six items showed low discrimination and evidence of differential item functioning (DIF). Only two items displayed DIF between individuals with low versus high psychopathy, while seven items showed DIF across country comparisons.

**Conclusion:**

Modifications are recommended, consistent with previous Latin American research, to further enhance the validity of the SRP-SF in forensic populations in this region.

## 1 Introduction

Psychopathy is a multidimensional personality construct that includes interpersonal (i.e., charming, grandiose, lying, manipulative), affective (i.e., remorselessness, shallow, callousness, fail to accept responsibility), behavioral (i.e., impulsive, irresponsible, parasitic, lack of goals, need for stimulation) and antisocial traits (Hare, 2003). Approximately 20% of the adult prison population exhibit elevated psychopathic traits, costing the criminal justice system billions (Kiehl and Hoffman, 2011). The Psychopathy Checklist-Revised (PCL-R; Hare, 2003) is the most widely used instrument for assessing psychopathic personality traits, particularly in forensic populations, due to its extensive validation and empirical applications (Hare and Neumann, 2008; Brodsky and Lichtenstein, 2020). However, it requires extensive time, specialized training, and access to collateral and clinical data, which limits its feasibility in complex contexts such as prisons (Boduszek and Debowska, 2016). To address these limitations, various self-report instruments have been developed and validated with forensic and non-forensic populations. These include not only a derivative of the PCL—R, the Self-Report Psychopathy-Short form (SRP-SF, Paulhus et al., 2016), but also the Triarchic Psychpathy Measure (TriPM; Patrick et al., 2009), the Levenson Primary and Secondary Psychopathy scales (LPSP; Levenson et al., 1995) and the Psychopathic Personality Inventory (PPI, Lilienfeld and Andrews, 1996).

The psychometric properties of the PCL-R and its self-report derivatives have been mainly examined using the framework of Classical Test Theory (e.g., Exploratory Factor Analysis [EFA]) and modern model-based assessment approaches, such as Confirmatory Factor Analysis (CFA). Despite ongoing debates about the PCL-R’s factor structure (Neumann et al., 2005), the four-factor model (Facet 1: Interpersonal, Facet 2: Affective, Facet 3: Lifestyle, and Facet 4: Antisocial) has been confirmed across various countries and populations (Neumann et al., 2015). Furthermore, preliminary evidence on the SRP-SF, using offender and non-offender samples from Europe and North America, supports its construct, convergent, and divergent validity, as well as its internal consistency and reliability (Boduszek and Debowska, 2016; Roy and Neumann, 2022). However, despite this growing evidence, traditional EFA and CFA approaches do not differentiate between respondent-level and item-level characteristics, which limits a more precise evaluation of how individual psychopathic traits function. This distinction is crucial, as traits like manipulation and deception may affect how individuals high in psychopathic traits respond to self-report measures (Ray et al., 2013). Therefore, further validation of the SRP-SF in Latin American forensic populations would benefit from using Item Response Theory (IRT) analysis.

IRT evaluates how well scale items represent a latent trait (e.g., psychopathy) and offers advantages over classical test methods (Tsang et al., 2018). By modeling the probability of responding to an item based on individual traits and item characteristics, it identifies items that effectively capture psychopathy facets and distinguish between different levels of psychopathy (Baker and Kim, 2017; Tsang et al., 2018). Additionally, Differential Item Functioning (DIF) explains the extent to which items measure psychopathy facets equivalently across different respondent groups (Baker and Kim, 2017), while the item–person map assesses the distribution of items across the underlying construct and evaluates potential measurement gaps or redundancies (Gordon, 2015). IRT studies on the PCL-R in justice-involved samples, primarily using Samejima’s (1969) graded response model (GRM), consistently show that Interpersonal (Facet 1) and Affective traits (Facet 2) better differentiate psychopathy levels, whereas Antisocial traits (Facet 4) display weaker precision (Cooke and Michie, 1997, 2001). However, methodological issues in Cooke et al.’s analyses (e.g., anchor selection) warrant caution (Bolt et al., 2007). More importantly, research with mega-samples (Hoppenbrouwers et al., 2015) has found that antisocial items exhibit strong discrimination parameters, highlighting the need to examine whether overt antisocial items add useful parametric information in self-report instruments with justice-involved samples.

The IRT framework has increasingly been applied to various self-report measures of psychopathy in general population samples such as the Short Dark Triad (Persson et al., 2017) and the Dirty Dozen (Webster and Jonason, 2013), the Triarchic Psychopathy Measure (Eichenbaum et al., 2021; Shou et al., 2018), the Levenson Psychopathy Scale (Gummelt et al., 2012), and the Psychopathic Personality Inventory (Eichenbaum et al., 2019). More comprehensive comparisons of item functioning within an IRT framework comparing several self-report measures have also been conducted (Tsang et al., 2018). The only study that applied IRT to an earlier version of the SRP-SF (i.e., SRP-II) found that cold-heartedness and disinhibition traits were less frequently endorsed than interpersonal and fearlessness traits among U.S. university students (Tsang et al., 2018). However, this sample is not comparable to justice-involved populations, and the SRP-II is a substantially different instrument. Thus, to the best of our knowledge, no study has used IRT to assess the SRP-SF in a non-Western forensic population (for an IRT study on a related instrument (the Psychopathy Checklist: Youth Version) in Brazil; see Ferraz et al., 2020).

The psychopathy construct measured with the PCL-R has been explored mainly in forensic populations from North America and some European countries, with sporadic studies conducted in Israel, Bulgaria, Russia, Hong Kong, Singapore, and Pakistan (see Fanti et al., 2018). Although these studies suggest that the psychopathy factor structure is similar across continents (Boduszek and Debowska, 2016; Neumann et al., 2015), some cultural differences have been reported in certain PCL-R indicators (for a review, see Fanti et al., 2018). Cultural differences have also been observed in self-report instruments such as the TriPM, where some items were not equivalent across Chinese and U.S. non-forensic samples (Shou et al., 2018). These findings highlight the importance of examining whether the functioning of psychopathy instrument items generalizes to other cultural settings, particularly in Latin America, where this type of research is limited and understanding item functioning is crucial for improving the validity of tools such as the SRP-SF (Roy and Neumann, 2022).

Latin America is classified as a non-WEIRD (non-Western, non-industrialized, non-rich, non-democratic) region in cross-cultural research frameworks (Henrich et al., 2010). At present, no study has applied IRT to the SRP-SF in a non-Western forensic population, despite its widespread use in North America and Europe. Our focus on prison samples from Chile and Uruguay addresses this gap. Although both countries are the only high-income nations in Latin America (Metreau et al., 2024), they share regional characteristics such as high levels of violence (with homicide rates of 6.4 in Chile and 11.3 per 100,000 in Uruguay in 2023; United Nations Office on Drugs and Crime, 2023) and weak investment in justice-involved individuals’ assessment and rehabilitation within their criminal justice systems. Despite the serious problem of violence in Latin America and the predictive value of psychopathy for violence and reoffending (Douglas et al., 2006; McCuish et al., 2015), research on psychopathy in the region remains limited (Folino, 2015), with only a few countries such as Chile, Brazil, and Uruguay reporting preliminary findings on prevalence and psychometric structure (Flores-Mendoza et al., 2008; León-Mayer et al., 2019; Trajtenberg et al., 2023; León-Mayer et al., 2014; Rocuant-Salinas et al., 2019). Correctional institutions in Latin America rarely assess psychopathic traits among inmates due to a lack of resources, limited trained staff, and scarce access to validated tools (Bergman and Fondevila, 2021). Analyzing the item functioning of the SRP-SF in these two non-Western forensic samples is crucial for evaluating the performance of quick and low-cost tools that can contribute to future assessment practices, sentence management, and rehabilitative efforts (Tew et al., 2014) in a region where risk assessment tools are underdeveloped.

This study expands the validation of the SRP-SF in Chile and Uruguay (Trajtenberg et al., 2023) by applying the IRT framework and testing item-level measurement across countries. Five objectives were addressed: (1) analyzing how well items discriminate between respondents (a parameter) and the level of psychopathy required for specific responses (b parameter); (2) assessing item functioning for potential misfit and response category usage; (3) identifying measurement gaps using the Wright map tool; (4) evaluating subscale-based information curves and item-level information for each trait; and (5) using Differential Item Functioning (DIF) to test country invariance and the influence of psychopathy levels on responses.

## 2 Methods

### 2.1 Participants and procedure

This study used archival samples (*N* = 539) from individuals who had previously given informed consent and completed the Spanish version of the SRP-SF (León-Mayer, 2012) as part of larger research projects. Sample 1 included 208 adult men in custody in Chile (mean age = 35.5, *SD* = 10.4), and Sample 2 included 331 male adults in custody from Uruguay (mean age = 31.27, SD = 9.06). In Chile, most participants were serving sentences for property-related offenses (e.g., theft, robbery, fraud), followed by crimes against persons (e.g., homicide, sexual assault) and a smaller group convicted of drug-related or other offenses (e.g., kidnapping). In Uruguay, violent offenses against persons were most common, with property offenses also represented and occurring more frequently than drug-related or other crimes (for sociodemographic details and additional information on criminal history, see Trajtenberg et al., 2023).

### 2.2 Measure

*Psychopathy*. The SRP-SF (Paulhus et al., 2016. Spanish adaptation by León-Mayer, 2012) consists of 29 total items divided into 4 scales: Interpersonal (7 items, *α* = 0.79 in Uruguay and α = 0.68 in Chile), Affective (7 items, α = 0.53 in Uruguay and α = 0.47 in Chile), Lifestyle (7 items, α = 0.76 in Uruguay and α = 0.67 in Chile), and Antisocial (8 items, α = 0.68 in Uruguay and α = 0.58 in Chile). Using a Likert scale, item responses range from 1 (“*Strongly Disagree”*) to 5 (“*Strongly Agree”*). Factor 1 assesses Interpersonal/Affective personality traits (e.g., *“I like scamming people,” “Never guilt over hurt people”*) and Factor 2 assesses Lifestyle/Antisocial traits (e.g., *“I rarely follow the rules,” “I have committed a serious crime”*). A higher score indicates higher psychopathic traits. The reliability for the global score was acceptable both in the Uruguayan (α = 0.89) and the Chilean sample (α = 0.85).

The SRP-SF instrument provides a strong alternative because it is based on the same four-factor model central to Hare’s conceptualization of psychopathy and has been widely used and validated across cultures (Neumann et al., 2012, 2015). Additionally, the SRP-SF’s brevity compared to other instruments offers a practical advantage, making it an efficient screening tool in countries or settings with limited financial resources and scarce staff trained in psychopathy assessment, such as many Latin American prisons. Evidence on the SRP-SF, using justice-involved and non-justice-involved samples from Europe and North America, supports its construct validity as well as its internal consistency (Boduszek and Debowska, 2016; Roy and Neumann, 2022). Previous research comparing the PCL-R and SRP-SF in justice-involved and community samples in the U.S. has shown a moderate association between both instruments and very similar four-factor psychometric structures (Neumann et al., 2015; Paulhus et al., 2016; Seara-Cardoso et al., 2020). Furthermore, the SRP-SF demonstrates robust associations with external covariates such as aggression, antisocial behaviors, and criminality (Debowska et al., 2014; Gordts et al., 2017; Neumann and Pardini, 2014), as well as with other self-report instruments of psychopathy such as TriPM (Carre et al., 2018).

### 2.3 Data analysis

The Graded Response Model (GRM; Samejima, 1969) was used because the items are polytomous (Debowska et al., 2018), and the scores for each item are consistently ordered, indicating that responses at the low end of the Likert scale reflect lower levels of psychopathy. A discrimination parameter (*a*) below 0.65 indicates low discrimination, whereas *a* > 1.34 and *a* > 1.69 are considered high and very high discrimination, respectively (Baker and Kim, 2017).

The Test Information Function (TIF) was calculated to assess how well the four facets estimate psychopathy across the trait range, and the Scale Information Curves (SIC) indicate the amount of information provided by each subscale across the latent trait continuum (*θ*). Curve peaks show where the test is most precise, while curve width reflects the range of psychopathy levels covered. Narrower curves indicate less precision at the extremes, while wider, flatter curves show broader coverage but lower precision. Interpretation of precision followed conventional guidelines based on the relation SE(θ) = 1/√I(θ) (Embretson and Reise, 2000): I < 3 indicates low precision, I around 5 suggests adequate precision, and I > 7–10 indicates very good precision.

Finally, the Wright (person–item) map was used to display the distribution of persons (left) and items (right) across the latent psychopathy construct for each facet. Inmates were ordered by their self-reported psychopathy levels, with lower levels at the bottom and higher levels at the top. Similarly, items were ranked, with harder-to-endorse items measuring higher psychopathy levels positioned higher, and easier items placed lower (Gordon, 2015). All analyses were conducted in RStudio (R Core Team, 2021) using the mirt (Chalmers, 2012), and WrightMap packages (Irribarra and Freund, 2014). R code and data are available upon request.

## 3 Results

Descriptive statistics of the SRP-SF for total and each facet are displayed in Table 1. The Facet 4 (Antisocial) yielded the highest score (*M* = 18.15, *SD* = 6.01), whereas the Facet 1 (Interpersonal) yielded the lowest score (*M =* 13.47, *SD* = 5.29).

**Table 1:** Descriptive statistics of the self-report psychopathy-short-form (SRP-SF).

Scale	Items	*M* (*SD*)	Min	Max	Item numbers
SRP—SF total	29	64.08 (18.19)	29	136	All items
Facet 1 interpersonal	7	13.47 (5.29)	7	35	SRP7, SRP9, SRP10, SRP15, SRP19, SRP23, SRP26
Facet 2 affective	7	16.14 (4.67)	7	31	SRP3, SRP8, SRP13, SRP16, SRP18, SRP24, SRP28
Facet 3 lifestyle	7	16.32 (5.89)	7	35	SRP1, SRP4, SRP11, SRP14, SRP17, SRP21, SRP27
Facet 4 antisocial	8	18.15 (6.01)	8	40	SRP2, SRP5, SRP6, SRP12, SRP20, SRP22, SRP25, SRP29

### 3.1 IRT

IRT analysis was conducted for each of the four psychopathy facets rather than for the composite measure of psychopathy, given previous evidence supporting the multidimensionality of this construct (Debowska et al., 2018). All 29 items were fitted to their corresponding facets in four graded response models for the total sample and the two country-specific samples. Parameter estimates and item fit statistics are displayed in Table 2.

**Table 2:** Graded response model item parameters.

Note	*Global*					Uruguay					Chile					
	a	b1	b2	b3	b4	a	b1	b2	b3	b4	a	b1	b2	b3	b4	DIF
Facet 1																
SRP7	1.80	0.37	1.21	1.41	2.05	2.29	0.29	1.04	1.17	1.67	1.16	0.50	1.56	1.92	2.87	0.40
SRP9	2.11	0.40	1.42	2.06	2.32	2.35	0.51	1.36	1.8	1.99	1.81	0.22	1.51	2.41	2.72	**0.00**
SRP10	1.76	0.12	1.02	1.46	2.36	2.00	0.21	0.92	1.36	2.06	1.41	0.02	1.24	1.71	2.97	0.03
SRP15	1.63	0.26	1.07	1.92	2.71	1.56	0.15	0.87	1.69	2.37	2.00	0.38	1.24	2.04	3.10	0.15
SRP19	2.08	0.04	0.86	1.42	2.07	2.18	0.05	0.82	1.24	1.86	1.48	−0.01	1.00	1.87	2.78	0.19
SRP23	1.33	−0.10	0.80	1.45	2.23	1.76	0.04	0.71	1.14	1.79	0.85	−0.36	0.99	2.11	3.21	0.02
SRP26	1.26	−0.28	0.63	1.57	2.82	1.16	−0.33	0.47	1.36	2.64	1.32	−0.25	0.81	1.8	3.19	0.61
Facet 2																
SRP3	0.31	−4.65	−1.75	1.54	6.35	0.42	−3.15	−1.23	1.15	4.59	0.11	−14.40	−4.89	3.53	17.6	0.40
SRP8	2.72	−0.04	0.57	1.09	1.78	2.99	−0.13	0.35	0.79	1.42	1.70	0.12	1.10	1.86	2.73	0.02
SRP13	0.23	2.24	5.65	7.11	9.79	0.28	2.61	5.55	6.92	8.40	0.49	0.51	2.21	2.83	4.61	**0.00**
SRP16	0.94	−0.90	0.14	0.83	2.33	1.18	−0.65	0.03	0.50	1.88	0.63	−1.52	0.49	1.68	3.52	0.91
SRP18	1.60	−0.71	0.06	0.75	1.80	1.81	−0.74	−0.13	0.31	1.37	1.09	−0.76	0.42	1.77	3.00	**0.00**
SRP24	0.64	0.57	2.37	3.66	4.50	0.57	0.52	2.28	3.53	4.66	0.99	0.46	1.91	3.05	3.43	0.72
SRP28	0.42	−0.16	1.70	2.66	5.24	0.24	−0.51	2.11	3.69	8.25	0.89	0.05	1.36	1.85	2.87	0.31
Facet 3																
SRP1	1.38	−0.62	0.11	1.18	2.21	1.60	−0.56	−0.01	0.78	1.76	0.88	−0.79	0.37	2.27	3.89	0.10
SRP4	1.44	−0.38	0.39	0.69	1.89	1.48	−0.57	0.08	0.31	1.56	1.23	−0.14	0.93	1.47	2.83	**0.00**
SRP11	1.96	−0.27	0.39	0.86	1.72	2.08	−0.22	0.29	0.76	1.61	2.40	−0.28	0.53	0.90	1.64	0.28
SRP14	1.08	−0.55	0.58	1.66	2.96	1.27	−0.34	0.53	1.28	2.31	0.86	−0.76	0.78	2.40	4.46	0.54
SRP17	1.22	−0.22	0.52	1.39	2.43	1.04	−0.56	0.13	0.99	2.31	1.25	0.22	1.19	2.27	3.10	**0.00**
SRP21	1.27	−0.39	0.41	0.95	1.86	1.49	−0.28	0.39	0.87	1.71	1.08	−0.49	0.46	1.00	2.03	0.06
SRP27	1.10	−0.27	0.75	1.45	2.97	1.22	−0.27	0.60	1.21	2.57	1.06	−0.23	1.04	1.77	3.23	0.70
Facet 4																
SRP2	0.13	−5.39	1.06	3.44	9.90	0.22	−2.52	0.92	2.43	6.24	0.23	−3.92	0.13	1.25	5.36	0.06
SRP5	1.51	−0.40	0.38	0.69	1.71	1.62	−0.42	0.25	0.46	1.50	1.40	−0.31	0.56	0.99	2.01	0.56
SRP6	1.45	0.40	1.29	1.45	2.13	1.50	0.34	1.08	1.24	1.83	1.05	0.61	1.97	2.26	3.52	0.61
SRP12	2.05	−0.09	0.41	0.59	1.34	2.15	−0.20	0.27	0.42	1.18	1.52	0.06	0.66	0.90	1.89	0.14
SRP20	0.23	−2.59	−0.63	0.22	3.09	0.54	−0.46	0.37	0.81	1.88	0.03	−40.01	−20.3	−14.76	15.51	**0.00**
SRP22	1.58	0.17	0.74	1.15	1.91	1.71	0.00	0.41	0.71	1.48	1.47	0.38	1.22	1.75	2.80	**0.00**
SRP25	1.90	0.30	0.79	1.03	1.73	2.03	0.22	0.62	0.82	1.54	2.01	0.29	0.92	1.19	1.83	0.83
SRP29	1.24	1.09	2.16	2.59	3.16	1.17	0.84	1.80	2.11	2.76	1.33	1.40	2.81	3.74	4.08	0.01

a = Estimated slope parameters; b1, b2, b3, and b4 = Estimated category threshold parameters; DIF = Differential Functioning Item are in bold (threshold alpha = 0.01).

In terms of discrimination parameters, values for the total sample ranged from 0.13 to 2.72. Six of the 29 items fell below the minimum threshold (0.64) and were therefore less informative, with SRP2 (*No co-offending*) and SRP13 (*Do not contact family*) being the least discriminant. These items, along with SRP16 (*Cold-hearted*), had low and flat information curves. Eight items showed very high discrimination (thresholds above 1.7), with SRP9 (*Scamming people*) and SRP8 (*Like to see fist fights*) being the most discriminant. Among the facets, Facet 2 (Affective) had the lowest average discrimination, including four less informative items. Facet 4 (Antisocial) had slightly higher discrimination with two less informative items, while Facet 3 (Lifestyle) had none. Facet 1 (Interpersonal) was the most discriminant, with no less informative items and all but two items being highly discriminant. Items measuring interpersonal aspects were the most effective at differentiating individuals with varying levels of psychopathy. Additionally, 11 items had b1 values above 0, indicating that even at low trait levels, endorsement required the latent trait to exceed 0.

### 3.2 Item functioning

**Figure 1:**
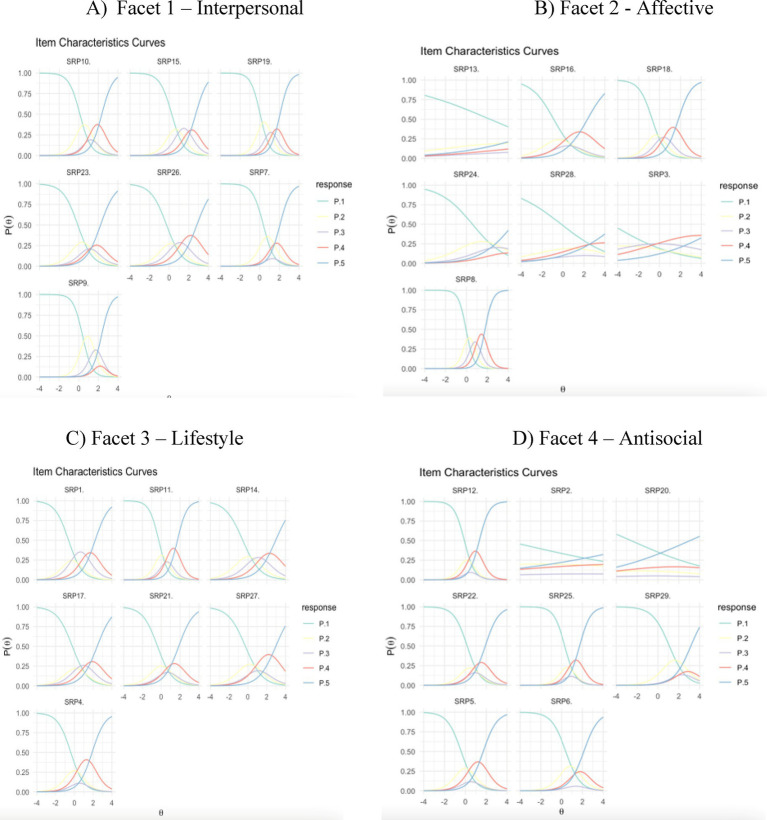
Item characteristic curves (ICC) for all items of the self-report psychopathy-short-form (SRP-SF) grouped by facet. **(A)** Facet 1—Interpersonal. **(B)** Facet 2—Affective. **(C)** Facet 3—Lifestyle. **(D)** Facet 4—Antoisocial.

Outfit/infit analysis showed that no items exhibited significant misfit (values above 1.5), and only one item (i.e., SRP8 *Like to see fist fights*) had low infit (below 0.5), suggesting it might not fully capture the range of the underlying trait. Additionally, some items displayed moderate infit/outfit values (below 0.89), indicating they might be degrading but still productive, particularly in Facets 3 (Lifestyle) and 4 (Antisocial), which contained three and four such items, respectively. Moreover, Figure 1 illustrates that higher psychopathy levels within a facet correspond to the selection of higher response categories. However, most items did not utilize all five options, with exceptions including SRP15 (*Take advantage before others*), SRP19 (*Pretend to like people to get things*), SRP26 (*People are easy to fool*), SRP8 (*Like to see fist fights*), SRP18 (*Love violent sports/movies*), and SRP14 (*Rarely follow rules*). Several items exhibited a dichotomous response pattern, with respondents primarily choosing “totally disagree” or “totally agree” and skipping intermediate categories [i.e., SRP6 (*Assaulted law enforcement staff*), SRP12 (*Broken into building*), SRP21 (*Get into trouble same things*), SRP22 (*Sometimes carry weapons*), SRP23 (*Get by telling people what want*), SRP25 (*Threaten people to get money*)].

**Figure 2:**
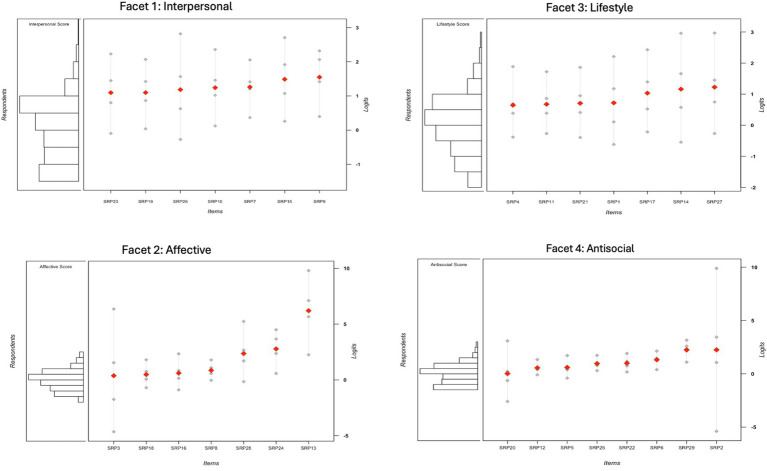
Wright map for each facet.

Figure 2 shows skewed distributions of persons and items across the four facets, with items clustered within a narrow range of the latent trait, especially in Facets 1 (Interpersonal) and 3 (Lifestyle). Only Facet 2 (Affective) demonstrated broad coverage, ranging from 0.37 to 6.2 on the latent trait continuum. Psychopathic traits were more widely distributed than items. However, no facet included individuals with extremely high scores, and the affective and antisocial facets lacked individuals with the lowest scores. The narrow item difficulty distribution suggests limited ability to differentiate across levels of affective traits. Facets 1 (Interpersonal), 3 (Lifestyle), and 4 (Antisocial) lacked coverage at both extremes, while Facet 2 (Affective) was especially limited in capturing lower scores, leaving gaps at trait extremes.

**Figure 3:**
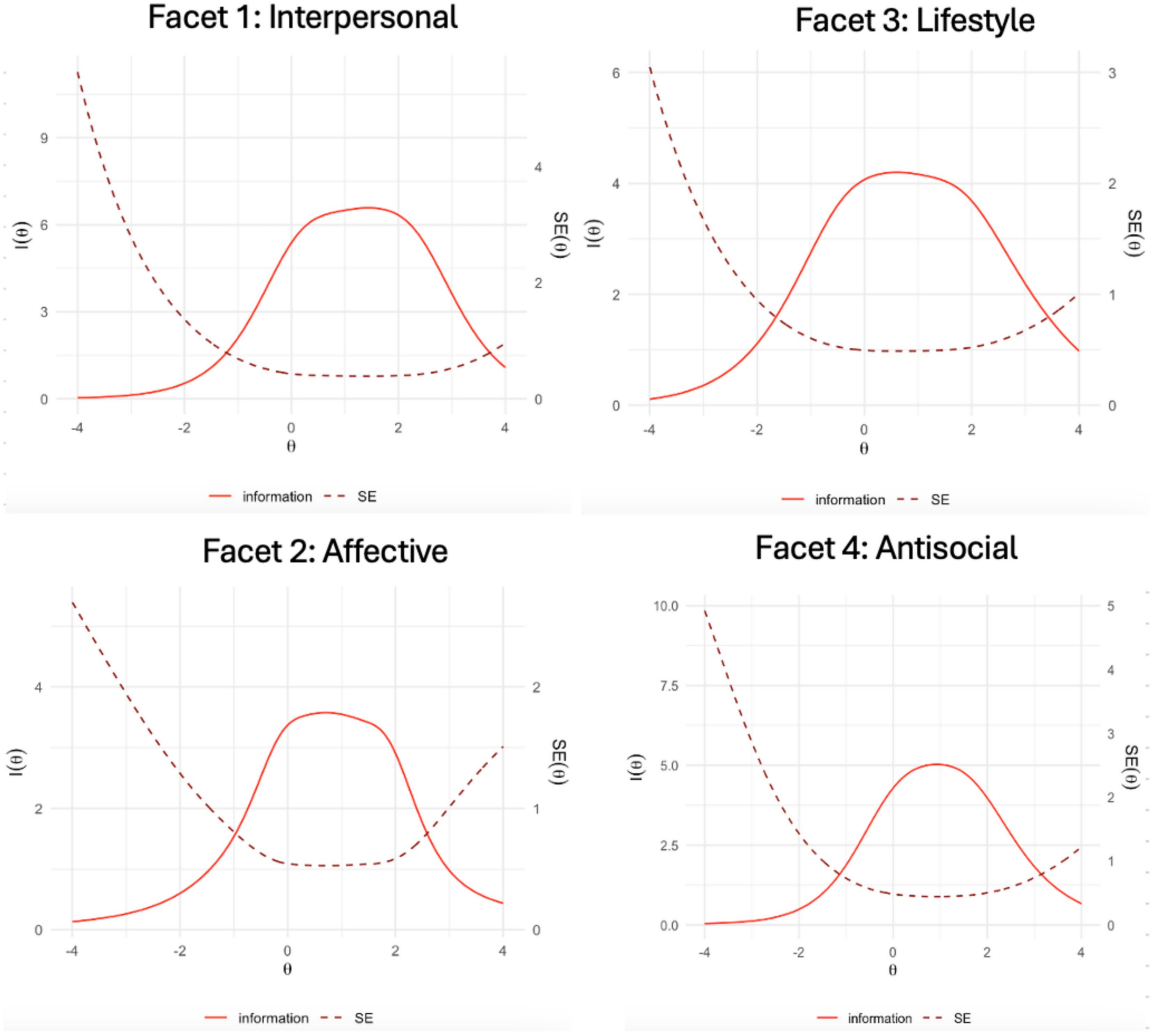
Test information items by facets.

No loops were observed, as all items captured all individuals and no items lacked corresponding persons, which typically occurs with overly easy or difficult items. Across facets, individuals’ mean psychopathy scores were below the mean item difficulty, with the smallest gap in Facet 4 (Antisocial, 0.9 SD) and the largest in Facet 1 (Interpersonal, 1.4 SD). Thus, on average, psychopathy items were socially unacceptable to endorse for many respondents, representing “very difficult’ items in IRT terms. The most difficult items were SRP9 (*Like scamming*), SRP13 (*Do not contact family*), SRP27 (*Mouth off without thinking*), and SRP2 (*No co-offending*) (Facets 1–4), while the easiest were SRP23 (*Get by telling people what want*), SRP3 (*Most people are wimps*), SRP4 (*Do dangerous things for thrill*), and SRP20 (*5 years or more sentence*) (Facets 1–4). Redundancy issues were noted in SRP10 (*Push people to break out*) and SRP7 (*Pretend to be someone to get*) (Facet 1), SRP11 (*Enjoy doing wild things*), SRP21 (*Get into trouble same things*), and SRP1 (*Rebellious*) (Facet 3), and SRP29 (*Try to hit people with vehicle*) and SRP2 (*No co-offending*) (Facet 4). Figure 3 shows varying precision across psychopathy levels, with all facets achieving their highest precision at moderate levels (approximately 0.5–1.5). Following standard interpretation guidelines (Embretson and Reise, 2000), TIF peaks indicate moderate to good precision for all facets. Facets 1 (Interpersonal) and 4 (Antisocial) were the most informative, each peaking above 5, while Facet 2 (Affective) was the least precise, peaking below 4 (see Figure 3). Curve widths also varied: Facets 1 (Interpersonal) and 3 (Lifestyle) provided broader coverage across the latent trait, indicating greater precision across a wider range of psychopathy levels. In contrast, Facet 2 (Affective) exhibited a narrower range, reflecting lower precision at both extremes of the trait.

### 3.3 DIF across countries and the SRP-SF

When comparing the samples of inmates from both countries, the Uruguayan sample showed higher item discrimination parameters than the Chilean sample, with seven highly and eight very highly discriminant items, compared to five each for Chile. In Uruguay, the least discriminant items were SRP2 (*No co-offending crimes*) and SRP13 (*No contact family*), while in Chile, SRP20 (*5 years or more sentence*) and SRP3 (*Most people are wimps*) were less effective at differentiating inmates with varying psychopathic levels. Both samples exhibited similar discrimination patterns across facets, consistent with the general sample. Moreover, DIF analysis revealed significant differences between the two samples: SRP9 (*Like scamming people*), SRP13 (*No contact family*), SRP18 (*Love violent sports/movies*), SRP4 (*Do dangerous for thrill*), SRP17 (*Sex with people you barely know*), SRP20 (*5 years or more sentence*), and SRP22 (*Sometimes carry weapons*) (see Table 1). Finally, to assess item invariance across different psychopathy levels, the sample was dichotomized using total psychopathy scale scores, with comparisons at the 75^th^ (top 25%) and 90th percentiles (top 10%). Only two items showed non-equivalence: SRP9 (*Like scamming*) at the 75th percentile and SRP7 (*Pretend to be someone else to get something*) at the 90th percentile.

## 4 Discussion

Understanding psychopathy, particularly in violent regions such as Latin America, is crucial due to its implications for legal decision-making and psychological treatment within the criminal justice system (Kiehl and Hoffman, 2011). Moreover, using brief and efficient assessment tools can inform sentence management and treatment planning in this context (Tew et al., 2014). Therefore, the aim of this study was to extend our knowledge about the SRP—SF in adult men in custody by examining item functioning and comparing results between Chile and Uruguay.

Our results contradict earlier studies that reported the highest discrimination in Facet 2 (Affective) using the PCL-R (Cooke and Michie, 1997, 2001). In contrast, we found that Facet 2 (Affective) showed the weakest discrimination (mean a = 0.98), while Facet 1 (Interpersonal) had the strongest (mean a = 1.71), followed by Facets 3 (Lifestyle) and 4 (Antisocial; mean a = 1.35 and 1.26, respectively). This aligns with recent findings suggesting that Facet 2 (Affective) provides the least discrimination between high and low PCL-R scores (Baglole et al., 2021; Mokros et al., 2011). Several factors may explain these differences in item efficiency including social context, cultural practices and sample type. For example, a study comparing Chinese and U.S. students found weaker Disinhibition and Meanness items in the Chinese sample using the Triarchic Psychopathy Measure (Shou et al., 2018). In our samples, SRP20 (*5 years or more sentence*) had the lowest discriminant power, possibly due to high punitiveness in Latin America, which is associated with longer sentences (Bergman and Fondevila, 2021). Regarding sample type, forensic samples are expected to show lower discrimination on some Facet 4 (Antisocial) items compared to general population samples (Neumann et al., 2015). Similarly, Facet 2 (Affective) items may have low discrimination not only due to emotional deficits in individuals but also because expressing emotions in prison is often costly or undervalued (Laws, 2018).

The Wright map findings indicated that very high and very low levels of psychopathy are not well detected by the SRP-SF, particularly in Facets 1 (Interpersonal) and 3 (Lifestyle), which was unexpected given the instrument’s goal of covering the entire latent construct. Although the SRP-SF was developed for the general population, previous studies suggest minimal qualitative difference between forensic and non-forensic samples (Boduszek et al., 2021). Our findings underscore the importance of identifying individuals with high psychopathy on Facets 1 (Interpersonal) and 3 (Lifestyle) in prison settings, as these individuals may struggle to engage in interventions yet still require them due to their elevated risk of reoffending. Including items to capture both high and low psychopathy levels is strongly recommended for use in these two countries.

Research consistently shows that respondents can fake psychopathic traits when instructed to do so, resulting in elevated scores on Dark Triad traits and related personality dimensions such as low self-control (For reviews, see Ray et al., 2013; Walker et al., 2022). However, this study found no significant response distortion among individuals with elevated psychopathic traits compared to those without, except for SRP7 (*Pretend to be someone else to get something*) and SRP9 (*Like scamming*), which showed non-equivalence. These findings align with a meta-analysis supporting the validity of self-report psychopathy scales (Ray et al., 2013), and a recent study reporting no link between response distortion and high psychopathy on the SRP4 (*Do dangerous things for thrill*) and SRP-SF, although the scale was more prone to overreporting, which may affect convergent validity (Knack et al., 2021). Future research should incorporate interview-based measures (e.g., PCL-R) and validity indicators to detect response distortion.

Finally, several items did not contribute to the accurate measurement of psychopathy in the two countries. Uruguay showed higher values on most items compared to Chile, except for seven items that were higher in Chile. However, previous research supports measurement equivalence, demonstrating strong invariance when comparing scalar and unconstrained models (Trajtenberg et al., 2023). These divergences may reflect differences in assumptions, model specifications, or sensitivity to minor item variations (Fischer and Karl, 2019). Although items generally perform similarly across countries (Roy and Neumann, 2022), modifications to certain items could improve the SRP-SF’s ability to detect psychopathy, as previously reported by (Rocuant-Salinas et al., 2019) in female offenders in Chile. Future studies should examine the impact of item modifications on the SRP-SF’s psychometric properties.

### 4.1 Limitations

The first limitation of this study is that the findings cannot be generalized to the prison population of Uruguay and Chile because the samples were not representative. Furthermore, our results cannot be generalized to forensic populations in other Latin American countries; therefore, replication in additional Latin American countries is encouraged. Second, we did not include indicators of behaviors or outcomes associated with psychopathy, which could provide additional evidence regarding item functioning. Future replications should incorporate external correlates and assess these traits not only in prison populations but also in the general population of both countries.

## 5 Conclusion

This study is the first to provide IRT analyses of the SRP-SF in two Latin American samples of adult men in custody. Overall, the SRP-SF identified psychopathy effectively but showed limited discrimination for detecting very high and very low levels of psychopathy. Although Chile had slightly lower item endorsement thresholds than Uruguay, overall country differences were small; however, modifying certain items may improve psychopathy identification in each context. These findings contribute to the growing literature on psychopathy in Latin America and, in particular, advance the validation of the SRP-SF in this region.

## Data Availability

The raw data supporting the conclusions of this article will be made available by the authors, without undue reservation.
